# Case Report: Reversible Neurotoxicity and a Clinical Response Induced by BCMA-Directed Chimeric Antigen Receptor T Cells Against Multiple Myeloma With Central Nervous System Involvement

**DOI:** 10.3389/fimmu.2021.552429

**Published:** 2021-02-25

**Authors:** Ying Zhang, Changfeng Zhang, Jin Zhou, Jingren Zhang, Xiaochen Chen, Jia Chen, Pu Wang, Xiuli Sun, Xiaoyan Lou, Wei Qi, Liqing Kang, Lei Yu, Depei Wu, Caixia Li

**Affiliations:** ^1^ Jiangsu Institute of Hematology, The First Affiliated Hospital of Soochow University, Suzhou, China; ^2^ Institute of Blood and Marrow Transplantation, Collaborative Innovation Center of Hematology, Soochow University, Suzhou, China; ^3^ Department of Cell Therapy, Livzon Mabpharm, Inc., Zhuhai, China; ^4^ UniCar Therapy, Ltd., Shanghai, China; ^5^ School of Chemistry and Molecular Engineering, East China Normal University, Shanghai, China

**Keywords:** central nervous system involvement, multiple myeloma, chimeric antigen receptor T cell therpay, neurotoxicity, case report

## Abstract

Isolated central nervous system involvement in multiple myeloma (CNS-MM) is rare and carries extremely poor prognosis. Chimeric antigen receptor T cell therapy (CART) targeting B-cell maturation antigen (BCMA) is demonstrated as a promising strategy in MM treatment, but the clinical safety and efficacy of BCMA-CART against isolated CNS-MM remain elusive. Here we report on a 56-year-old male with refractory isolated CNS-MM who received autologous BCMA-CART therapy and developed grade 4 neurological complications. Cerebrospinal fluid (CSF) analyses showed significant expansion of CART cells and a substantially elevated interleukin-6 (IL-6) level. Intravenous methylprednisolone was administered and the symptoms resolved gradually. Unexpectedly, the level of IL-6 in the CSF was maintained for another 3 days even after the relief of the neurological symptoms. A partial response was achieved and sustained for 5.5 months. This is the first report describing a patient with isolated CNS-MM treated using BCMA-CART therapy. The results demonstrated that BCMA-CART cells administered intravenously trafficked into the CSF, eradicated tumor cells, and induced severe but reversible neurological adverse events. This single-patient report suggests that BCMA-CART therapy can be considered as an alternative option for isolated CNS-MM.

**Clinical Trial Registration:**

ClinicalTrials.gov, identifier NCT03196414.

## Introduction

Multiple myeloma with central nervous system involvement (CNS-MM) is extremely rare and incurable with very limited treatment options. CNS-MM accounts for less than 1% of the total MM population and is characterized by disease-associated neurological symptoms, computed tomography (CT) and/or magnetic resonance imaging (MRI) abnormalities and presence of atypical plasma cells in patients’ cerebrospinal fluid (CSF) (1). Current therapeutic strategies for CNS-MM consist of systemic and/or intrathecal chemotherapy, radiotherapy, and autologous or allogeneic stem cell transplant, but the prognosis is unsatisfying (1, 2).

Chimeric antigen receptor T cell therapy targeting B-cell maturation antigen (BCMA-CART) is a potential therapeutic strategy for CNS-MM. BCMA-CART therapy exhibited robust antitumor activity against systemic MM in several clinical trials (3, 4). Additionally, CART cells administered intravenously were demonstrated to be able to transfer across the blood brain barrier (BBB) and elicit responses in patients with both hematological and solid CNS malignancies (5–7). However, patients with CNS-MM were excluded from most CART trials because of the concern for life-threatening neurological complications, and thus information about CART therapy against CNS-MM is scarce.

Here we report on a single patient with isolated CNS-MM from an ongoing BCMA-CART trial. Following CART infusion, this patient experienced grade 4 neurotoxicity which resolved with prompt recognition and treatment using methylprednisolone and mannitol. A partial response was observed which was sustained for 5.5 months. This is to our knowledge the first report regarding a patient with isolated CNS-MM treated by CART therapy. This report also describes the cytological and biochemical changes in the patient’s CSF in various post-infusion time points. The clinical course suggests that CNS-MM can be treated by BCMA-CART therapy if the toxicity is managed appropriately.

## Case Description

**Figure 1:**
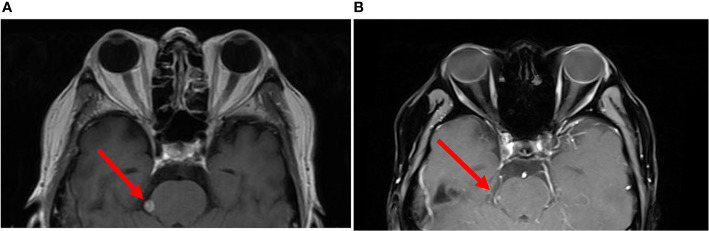
Brain imaging of the patient before and after CART treatment. **(A)** Brain MRI immediately before CART infusion. **(B)** The same region on day 30 after CART infusion. Red arrows indicate the location of one of the intracranial abnormalities. The patient was discharged on day 24, and panel **(B)** was done using a different MRI instrument in a local hospital in the patient’s hometown.

A 56-year-old male was diagnosed with *κ* light chain MM in 2014 for which he received five lines of therapy including proteasome inhibitors and immunomodulatory drugs (**Supplementary Table 1**). Disease involvement of CNS and spinal cord was discovered in 2017 and was characterized by impaired right vision, limited neck mobility, and detection of monoclonal plasma cells on CSF analysis. The patient then received another three lines of therapy, but the CNS/spine lesions were refractory to treatment including combinations of daratumumab, cytarabine, methotrexate, dexamethasone, cis-platinum, etoposide, and cyclophosphamide. He was then enrolled in a clinical trial of BCMA-CART therapy against MM (NCT03196414, currently recruiting, **Supplementary Method**). Immediately prior to CART treatment, the patient was confirmed to have stage I refractory CNS-MM according to the Revised International Staging System for multiple myeloma (R-ISS). The baseline findings at study enrollment included visual impairment, bilateral paraplegia, bladder and bowel incontinence, an elevated κ/*λ* ratio on CSF analysis (**Supplementary Table 2**), and a MRI result showing a leptomeningeal lesion of 6.8 × 5.8 mm (**Figure 1A**) and several other small abnormalities of the right occipital lobe and thoracolumbar spine. Subjectively, the patient complained of lower back stiffness and lower extremity weakness and indicated no history of cognitive disorder or seizure. A bilateral bone marrow biopsy immediately before CART treatment indicated no marrow disease, suggesting the patient had isolated CNS-MM.

**Figure 2:**
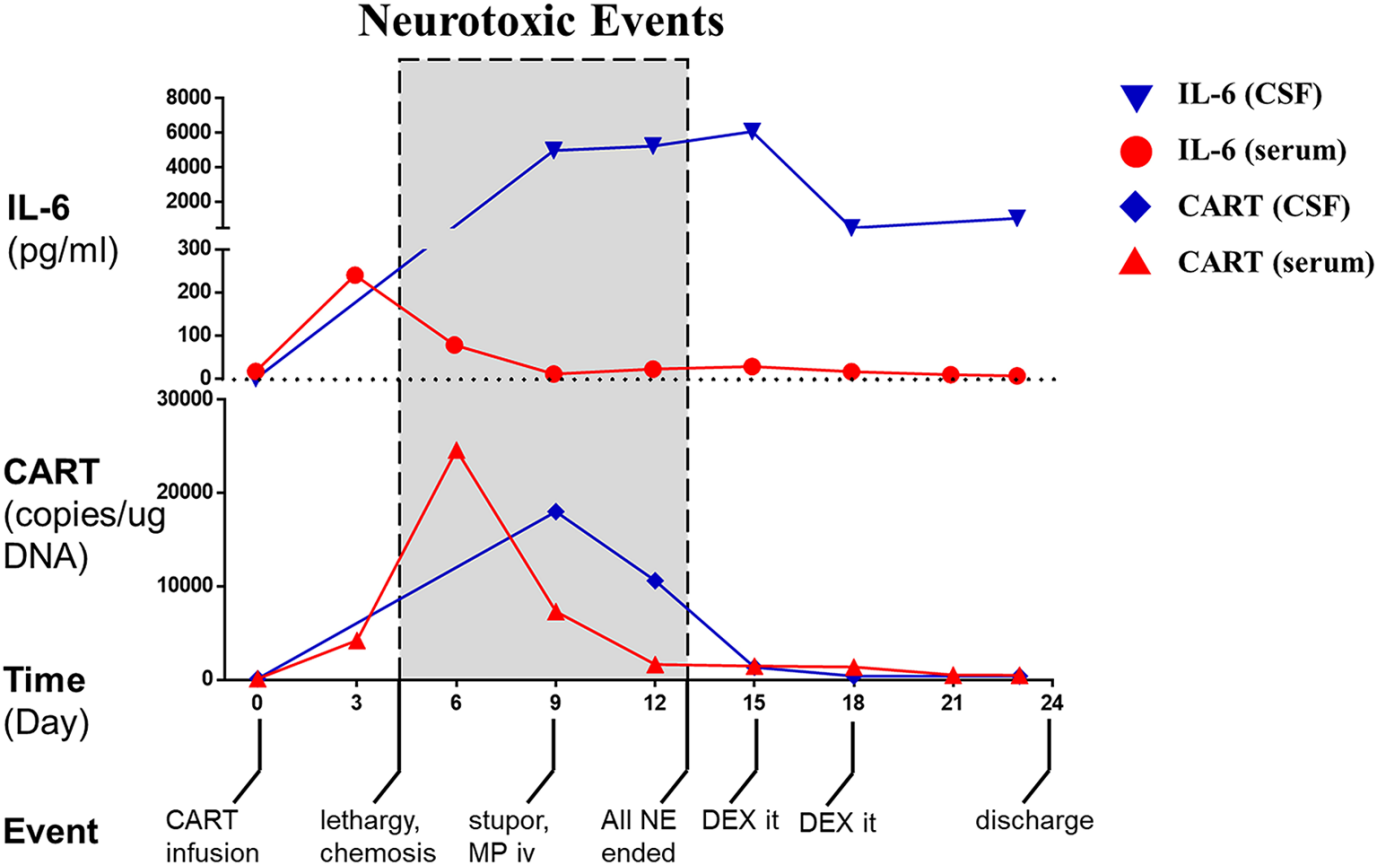
CART cells, IL-6 and important events of the patient before and after CART treatment. The level of IL-6 in the peripheral blood (serum, dot) and cerebrospinal fluid (CSF, nabla) and the number of CART cells in the peripheral blood (serum, triangle) and cerebrospinal fluid (CSF, diamond) were curved over time. Shadow boxes show the period when neurotoxic events were observed. NE, neurotoxic event; MP, methylprednisolone; DEX, dexamethasone.

On our phase I trial, the patient received one intravenous infusion of 5 × 10^7^/kg autologous BCMA-CART cells per body weight without prior lymphodepletion (**Supplementary Method**). Lymphodepleting chemotherapy was not administered due to the concerns of his lymphopenia (absolute lymphocyte count at study enrollment: 0.25 ×10^9^/L) and an increased risk of neurotoxicity (8). Six hours after the infusion, he developed fever and hypotension (**Supplementary Table 3**). The patient’s neurological status was monitored daily in a similar way to the CD19 CART criteria (9). Two days later, the patient complained of headache but remained awake and alert. On day 4, the patient developed lethargy and obtundation (responded slowly and hesitantly to verbal stimuli), and chemosis and stiff neck were also observed. His neurological symptoms further progressed on day 9 manifested as stupor, aphasia (could not give any verbal response), pupil asymmetry and loss of light reflex (left: 3 mm, normal reflex; right: 5 mm, no reflex), assessed as grade 4 neurotoxic events. To further evaluate these neurological findings, a CSF analysis was performed. The results revealed that interleukin-6 (IL-6, **Figure 2**) had increased from 10 pg/L measured before CART treatment to 5,000 pg/L accompanied by slight increases in interferon *γ*, IL-2, and IL-10 (**Supplementary Table 4**). Other significant results included pleocytosis and high levels of lactate dehydrogenase (LDH) and protein (**Supplementary Table 2**). Furthermore, a contemporaneous CAR transgene analysis showed a significant number of CART cells in his CSF on day 9 post-infusion (**Figure 2**). Taken together, these results indicated that the neurological symptoms were probably caused by the CSF CART cells. The patient therefore received intravenous methylprednisolone (40 mg, single dose) and mannitol, and the neurological symptoms improved gradually. Tocilizumab was not used considering that it does not penetrate the BBB and because its use was not warranted in the absence of systemic symptoms (**Supplementary Table 3**). By day 13, lethargy disappeared, and pupils returned to normal. Interestingly, dramatic levels of IL-6 continued to be detected in his CSF at various time points. Therefore, he received intrathecal dexamethasone (5 mg) on day 15 and day 18 which was followed by a significant drop in the CSF IL-6 level. By day 24, the patient exhibited a normal mental status with rapid and accurate responses to verbal questions. A favorable response of the disease was observed indicated by a reduction of more than 50% of the leptomeningeal lesion by MRI on day 30 (**Figure 1B**), disappearance of monoclonal plasma cells and a normal *κ*/*λ* ratio in his CSF on day 23 (**Supplementary Table 2**), which was assessed as a partial response per International Myeloma Working Group (IMWG) criteria (**Supplementary Method**). The patient indicated regaining of his urinary continence and had slight improvements of lower limb movement at month 1 and month 3 follow-ups. His clinical response persisted through 5.5 months.

## Discussion

CNS-MM carries a very poor prognosis. There are no standard treatment guidelines, and current strategies include systemic therapy, intrathecal therapy, and CNS irradiation alone or in combination. However, most of the approaches are either poor at penetrating the BBB (such as cyclophosphamide and bortezomib) or of limited benefit (such as thalidomide), and there is a paucity of data to provide clear evidence of efficacy for new drugs such as pomalidomide and marizomib. Stem cell transplantation has shown good efficacy against CNS-MM but is not considered in many patients due to their short survival time (10). Therefore, novel strategies such as BCMA-CART therapy have a high potential.

Several concerns exist regarding BCMA-CART therapy against CNS-MM. A major concern is whether BCMA-CART cells transfer across the BBB in sufficient numbers. In prior studies, CART cells targeting CD19 or epidermal growth factor receptor variant iii (EGFRvIII) administered intravenously were detected in patients’ CSF (5–7, 11, 12). Activated T cells with no gene-editing can transfer across the BBB mediated by molecules such as intercellular adhesion molecule 1 (ICAM-1) and vascular cell adhesion molecule 1 (VCAM-1), and thus it is not surprising that CART cells behave the same way (13). In this report, we show that BCMA-CART cells could transfer effectively across the BBB. Although their kinetic profiles in the CSF were comparable to CART cells in the peripheral blood, it took 9 days to reach the maximal CAR transgene number in the patient’s CSF compared with 6 days in his peripheral blood. We speculate that this difference might be caused by CART penetration of the BBB, distribution within the CNS, and expansion upon encountering BCMA-positive tumor cells.

Another concern is increased risk of neurotoxicity. Neurotoxicity has been reported in several trials using BCMA-CART therapy against MM, but the etiological nature is elusive (3, 14). In early CART trials against leukemia or lymphoma undetected low-level expressions of target antigens in CNS tissues had been once viewed as the cause of neurotoxicity, while later results indicated that BBB dysfunction due to systemic inflammation was the primary culprit (5, 6, 15). Some pre-clinical results revealed that multiple cytokines, such as IL-6 and interleukin-1 (IL-1), induced neurotoxicity synergistically (16, 17). Other reports indicated that cytokines produced in CNS played an important role in neurotoxicity (18). However, the etiological factors for patients with CNS-involved hematological malignancies are not well defined as all the reported patients had both CNS and marrow disease (12). Therefore, it has been difficult to elucidate whether these neurological complications were independent local events, or consequences of the systemic immune responses.

In our report, a patient with isolated CNS-MM received BCMA-CART therapy and developed severe neurological complications. Our results suggested that CNS-infiltrated CART cells induced the neurological complications independently because the patient had no marrow disease and a low CAR transgene number in the peripheral blood during the period of neurotoxicity. Additionally, a substantial difference in IL-6 level between time-matched CSF and serum samples indicated that the inflammatory responses were largely localized to the CSF. Additionally, cytological results indicated that multiple types of white blood cells infiltrated into the CSF, reminiscent of a report revealing that monocyte-lineage cells could be recruited by CART-tumor interaction and produce pro-inflammatory cytokines such as IL-6 (17). These results were also major considerations when we decided not to give the patient tocilizumab for neurotoxicity. Specifically, the results indicated that the symptoms were caused by a series of local events mostly restricted to his CSF (9). We would have considered adding tocilizumab to his management if the symptoms had been derived from systemic inflammatory responses with evidence of endothelial activation and increased BBB permeability (15). The neurological symptoms were relieved by methylprednisolone, while it is also possible that those symptoms were self-limiting (14). Our observation was inconsistent with a previous report demonstrating methylprednisolone did not work against CNS toxicities, which might be due to the variability between underlying mechanisms (19).

Interestingly, the IL-6 level in the CSF was sustained for several days after the drop in CART cell number and the resolution of neurotoxicity. The temporal inconsistency between the IL-6 level and CART cell number suggested that IL-6 might be secreted at first by CART cells and then by other leukocytes in the CSF considering that the IL-6 level and total white blood cell count were sustained and dropped simultaneously. Moreover, the CSF cytological analyses indicated a switch of the predominant cell type from neutrophil to monocyte, which was somewhat similar to the neutrophil-monocyte transition in an acute phase response (20). The temporal inconsistency between the IL-6 level and neurotoxicity suggested that a reduction in IL-6 level was not a prerequisite for resolution of neurotoxicity in our patient. This inconsistency might occur through two possible mechanisms: (a) the amount of soluble IL-6 receptor, an indispensable agonist of IL-6 signaling, might be insufficient to trigger the IL-6 pro-inflammatory pathway leading to an apparently high IL-6 level; (b) other factors besides IL-6 (such as IL-1) played certain roles and thus the IL-6 level alone could not reflect the regression of neurotoxicity.

There are several limitations to this single patient report. CAR transgene number, IL-6 level and other biomarkers in the patient’s CSF were not measured in the first a few days considering the risk of lumbar puncture, and thus the early changes were not available. Moreover, IL-6 associated factors, such as soluble IL-6 receptor and soluble gp130, were not assessed, and a whole picture of IL-6 signaling was not demonstrated. Lastly, few investigations were done regarding the post-CART relapse and it was hard to determine the potential causes of the relapse such as poor CART persistence or loss of BCMA expression in tumor cells.

## Concluding Remarks

We describe a rare patient with refractory isolated CNS-MM who was treated using CART cells targeting BCMA leading to a partial response that was sustained for 5.5 months. The patient developed severe neurotoxic events distinct from the symptoms of the primary disease probably resulting from the CART therapy, which was reversed following glucocorticoid treatment. Biomarker analyses indicated infiltrations of multiple types of immune cells and an inconsistency between the neurotoxic events and elevated pro-inflammatory cytokine IL-6. Taken together, this report suggests the feasibility of effective treatment of CNS-MM with CART cells administered intravenously, highlights the uses of methylprednisolone and mannitol for neurotoxicity management, and emphasizes necessities of further investigations of neurotoxicity caused by the interaction of CNS local lesions and infiltrated CARTs.

## Data Availability Statement

The raw data supporting the conclusions of this article will be made available by the authors, without undue reservation.

## Ethics Statement

The studies involving human participants were reviewed and approved by Institutional Ethics Committee of the First Affiliated Hospital of Soochow University. The patients/participants provided their written informed consent to participate in this study. Written informed consent was obtained from the individual(s) for the publication of any potentially identifiable images or data included in this article.

## Author Contributions

YZ was the physician-in-charge. CZ designed the clinical protocol, analyzed data and wrote the manuscript. JZ, JRZ, XC, and JC were the clinicians who participated in the treatment of the patient. XS, XL, WQ, LK, and LY designed and manufactured the CART cells. DW was the principal investigators of the trial. CL was the secondary investigators of the trial. YZ, CZ, DW, and CL contributed equally to this study. All authors contributed to the article and approved the submitted version.

## Funding

National Natural Science Foundation of China (81730003), National Science and Technology Major Project (2017ZX09304021), and Science Planning Project of Suzhou (sys2018049) provided grant to support the study.

## Conflict of Interest

CZ is an employee of Livzon Mabpharm, Inc and UniCar Therapy Ltd. XL and XS are employees of UniCar Therapy Ltd.

The remaining authors declare that the research was conducted in the absence of any commercial or financial relationships that could be construed as a potential conflict of interest.

## Abbreviations

BBB, blood brain barrier; BCMA, B-cell maturation antigen; CART, chimeric antigen receptor T; CNS, central nervous system; CSF, cerebrospinal fluid; CT, computed tomography; EGFRvIII, epidermal growth factor receptor variant iii; ICAM-1, intercellular adhesion molecule 1; IL-1, interleukin-1; IL-6, interleukin 6; IMWG, international myeloma working group; LDH, lactate dehydrogenase; MM: multiple myeloma; MRI, magnetic resonance imaging; R-ISS, revised international staging system; VCAM-1, vascular cell adhesion molecule 1.
