# Supplementary material for: Case Report: Reversible Neurotoxicity and a Clinical Response Induced by BCMA-Directed Chimeric Antigen Receptor T Cells Against Multiple Myeloma With Central Nervous System Involvement
Source: Front Immunol. 2021 Feb 25;12:552429. doi: 10.3389/fimmu.2021.552429 (PMC7947195; doi:10.3389/fimmu.2021.552429)
Supplement: Supplementary file 1 [file DataSheet_1.docx]

**Supplemental Table 1. Lines of therapies prior to CART therapy**

| Line | Regimen | Administration route | Response |
| --- | --- | --- | --- |
| 1 | Bortezomib, pharmorubicin, dexamethasone |  | VGPR |
| 2 | Autologous stem cell transplant |  | sCR |
| 3 | Lenalidomide, dexamethasone, clarithromycin |  | sCR |
| 4 | Bortezomib, cyclophosphamide, dexamethasone |  | sCR |
| 5 | Bortezomib, lenalidomide dexamethasone |  | sCR |
|  | *Diagnosed as CNS-involved disease* | | |
| 6 | Cytarabine, methotrexate, dexamethasone | Intrathecal | SD |
| 7 | Cis-platinum, etoposide, cyclophosphamide, dexamethasone |  | SD |
|  | Cytarabine, methotrexate, dexamethasone | Intrathecal |  |
| 8 | Daratumumab |  | PD |
|  | Daratumumab, cytarabine, methotrexate, dexamethasone | Intrathecal |  |
| VGPR: very good partial response; sCR: stringent complete response; SD: stable disease; PD: progressive disease | | | |

**Supplemental Table 2. Pertinent results of cerebrospinal fluid analysis**

|  |  | Before Treatment ^a^ | Day 9 ^c^ | Day 12 | Day 15 ^d^ | Day 18 ^d^ | Day 23 |
| --- | --- | --- | --- | --- | --- | --- | --- |
| Cell count (cells/mm^3^) | | | | | | | |
|  | Total WBC | 2 | 340 | 247 | 230 | 48 | 96 |
|  | Monocytes | 1 | 10 | 41 | 81 | 39 | 86 |
|  | Polymorphonuclear neutrophils | 1 | 330 | 206 | 149 | 9 | 11 |
| Biochemicals | | | | | | | |
|  | Protein (g/L) | 0.20 | 0.74 | 1.36 | 1.24 | 0.85 | 0.96 |
|  | LDH (U/L) | 9 | 167 | 110 | 67 | 69 | 53 |
|  | IL-6 (pg/L) | ND | 5000 | 5256 | 6091 | 525 | 1076 |
| Tumor associated factor | | | | | | | |
|  | κ/λ ratio | 64.3 | NA | NA | 1.3 | NA | NA |
| 1. Data from the closest tests before CART infusion 2. Measured by qPCR 3. Methylprednisolone administrated intravenously 4. Dexamethasone administrated intrathecally   ND: not detected; NA: no data available | | | | | | | |

**Supplemental Table 3. List of systemic adverse events**

| AE | Grade ^a^ | Start ^b^ | Stop ^b^ | Note |
| --- | --- | --- | --- | --- |
| Low hemoglobin count | 3 | n/a ^c^ | Day 24 |  |
| Thrombocytopenia | 3 | n/a ^c^ | Day 7 |  |
| Fever | 3 | Day 0 | Day 19 | Peak temperature: 39.8℃, Day 1; NSAIDs, external cooling and prophylactic antibiotics were used |
| Hypotension | 2 | Day 0 | Day 4 | No vasopressor used |
| Elevated ALT/AST/γ-GGT | 2 | Day 1 | Day 11 |  |
| Hypoxemia | 2 | Day 4 | Day 21 | Oxygen delivered by nasal cannula |
| Diarrhea | 1 | Day 11 | Day 13 |  |
| 1. According to CTCAE v 5.0 2. Event timing is provided as time since infusion in days 3. n/a indicated the adverse events existed before CART treatment   NSAID: non-steroidal anti-inflammatory drug; ALT: alanine transaminase; AST: aspartate transaminase; γ-GGT: gamma-glutamyl transpeptidase | | | | |

**Supplemental Table 4.** **Other cytokine levels of the patient**

|  |  | Baseline value (pg/ml) | Peak value (pg/ml) | Peak time ^a^ |
| --- | --- | --- | --- | --- |
| IL-2 | serum | ND | ND | n/a |
|  | CSF | ND | 38 | Day 12 |
| IL-4 | serum | ND | 11 | Day 9 |
|  | CSF | ND | ND | n/a |
| IL-10 | serum | ND | 13 | Day 3 |
|  | CSF | 10 | 81 | Day 15 |
| TNF-α | serum | ND | 6 | Day 9 |
|  | CSF | ND | 7 | Day 12 |
| IFN-γ | serum | ND | 236 | Day 3 |
|  | CSF | ND | 193 | Day 15 |
| IL-17A | serum | ND | ND | n/a |
|  | CSF | ND | ND | n/a |
| ND: not detected; n/a: not available; IL: Interleukin; IFN-γ: interferon γ; TNF-α: tumor necrosis factor α; CSF: cerebrospinal fluid.  a. Timing is provided as time since infusion in days | | | | |

**Supplementary Method**

*Clinical protocol.* The patient was enrolled into a CART trial (NCT03196414) with the approval of the Institutional Ethics Committee of the First Affiliated Hospital of Soochow University. Treatment consisted of a single infusion of BCMA-directed 3rd generation CART cells equipped with CD28 and OX40 co-stimulatory domains and CD3ζ domain (5x10^7^ CAR-positive cells per kilogram bodyweight) following pre-conditioning chemotherapy consisting of fludarabine (30 mg/m^2^, 3 days) and cyclophosphamide (300 mg/m^2^, 3 days). The inclusion criteria included (a) BCMA antigen expression; (b) relapsed and/or refractory multiple myeloma; (c) expected survival ≥ 3 months; (d) baseline conditions: creatinine < 2.0 mg/dl, PT and APTT < 2x normal, arterial blood oxygen saturation > 92%; alanine aminotransferase/aspartate aminotransferase < 3x normal value, Karnofsky scores ≥ 60 and ECOG score ≤ 2; (e) adequate venous access for apheresis; (f) no system chemotherapy in one month and immunotherapy in three months prior to CART infusion. The exclusion criteria included (a) pregnant or lactating women; (b) uncontrolled active infection; (c) active hepatitis B or hepatitis C infection; (d) concurrent use of systemic steroids; (e) previously treatment with any gene therapy products; (f) any uncontrolled active medical disorder that would preclude participation; (g) HIV infection; (h) a history of myocardial infarction and severe arrhythmia in half a year; (i) any form of primary immunodeficiency (such as severe combined immunodeficiency disease); (j) patients with fever of unknown origin. The primary objective was to determine the incidence of grade 3 or higher adverse events associated with CART cells. The secondary objective included (a) overall response rate and (b) in vivo expansion and persistence of CART cells. Grading for neurotoxicity and other adverse events were performed by the treating physician per ASTCT Consensus Grading and CTCAE v5 (1). Response assessments for regular patients were determined by International Myeloma Working Group response criteria (2). CART expansion and persistence were assessed by measuring CAR transgene copy numbers in peripheral blood and CSF using quantitative polymerase chain reaction.

For the patient in this report, written informed consent was obtained and peripheral blood mononuclear cells were collected by leukapheresis for BCMA-CART generation. Pre-conditioning chemotherapy was canceled after discussions among the principal investigator and treating physicians considering the specific conditions of the patient, and this alteration was reported to the Institutional Ethics Committee of the First Affiliated Hospital of Soochow University.

*CAR construct.* The CAR construct consisted of a murine-derived anti-BCMA single-chain fragment variable (clone number C11D5.3), a CD8 transmembrane domain, the signaling domains of CD28, OX40 and CD3ζ.

*CAR-T manufacture.* Autologous T cells were isolated from apheresis blood by gradient centrifugation and enriched using anti-CD3 magnetic beads (Miltenyi). T cells were then stimulated with anti-CD3 (Miltenyi) and anti-CD28 (Miltenyi, catalog 170-076-117) monoclonal antibodies, and transduced with lentiviral vectors encoding BCMA-specific CARs. CAR-T cells were cultured in AIM-V media (Gibco) supplemented with 10% autologous human serum, 100 IU/ml IL-2 (PeproTech), 5 ng/ml IL-7 (PeproTech), and 5 ng/ml IL-15 (PeproTech) for 9-12 days.

*Cytokine measurement*. IL-6 levels in serum and CSF at various time points were detected using Th1/Th2 Cytometric Bead Array kits (BD) following the manufacturer’s protocol.

*CAR transgene copy number measurement.* Quantitative polymerase chain reaction was used in the quantifications of CAR transgene copy numbers. Briefly, mRNA of CART cells was extracted using One-Step RT–PCR Kit (Qiagen) and converted to cDNA. Reverse transcripts were amplified on an ABI-7500 system (Life Technology). CAR copy numbers were detected targeting the Woodchuck Hepatitis Virus Posttranscriptional Regulatory Element sequence which was part of the CAR construct (3).

Reference

[1] Lee DW, Santomasso BD, Locke FL, Ghobadi A, Turtle CJ, Brudno JN, et al. ASTCT Consensus Grading for Cytokine Release Syndrome and Neurologic Toxicity Associated with Immune Effector Cells. *Biol Blood Marrow Transplant.* (2019) 25:625-638.

[2] Kumar S, Paiva B, Anderson KC, Durie B, Landgren O, Moreau P, et al. International Myeloma Working Group consensus criteria for response and minimal residual disease assessment in multiple myeloma. *Lancet Oncol.* (2016) 17:e328-e346.

[3] Wang X, Popplewell LL, Wagner JR, Naranjo A, Blanchard MS, Mott MR, et al. Phase 1 studies of central memory-derived CD19 CAR T-cell therapy following autologous HSCT in patients with B-cell NHL. *Blood*. (2016) 127:2980-90.
